# Supplementary material for: OMNI-P2x universal neural network potential for excited-state simulations
Source: Nat Commun. 2026 Apr 7;17:4949. doi: 10.1038/s41467-026-71380-5 (PMC13233842; doi:10.1038/s41467-026-71380-5)
Supplement: Supplementary file 1 — Supplementary Information [file 41467_2026_71380_MOESM1_ESM.pdf]

Supporting Information for "OMNI-P2x Universal Neural Network Potential for Excited-State Simulations"

Mikolaj Martyka[1], Xin-Yu Tong[2], Joanna Jankowska[⋆,1], and Pavlo O. Dral[⋆,2,3,4,5]

[1]Faculty of Chemistry, University of Warsaw, Pasteura 1, Warsaw, 02-093, Poland
[2]State Key Laboratory of Physical Chemistry of Solid Surfaces, College of Chemistry and Chemical Engineering, and Fujian Provincial Key Laboratory of Theoretical and Computational Chemistry, Xiamen University, Xiamen, Fujian 361005, China
[3]Institute of Physics, Faculty of Physics, Astronomy, and Informatics, Nicolaus Copernicus University in Toruń, ul. Grudziadzka 5, 87-100 Toruń, Poland
[4]Institute of Advanced Studies, Nicolaus Copernicus University in Toruń, ul. Wileńska 4, 87-100 Toruń, Poland
[5]Aitomistic, Shenzhen 518000, China

Contents

1 Fragment correction scheme **3**

2 Performance of OMNI-P2x **6**
2.1 Error statistics of excitation energies 6
2.2 Predicted spectra 7
2.3 Non-equilibrium structures 8

3 High-throughput screening of azobenzene derivatives **11**

4 Nonadiabatic molecular dynamics **12**
4.1 Fulvene 12
4.2 Azobenzene 15

5 Performance of B3LYP **18**

6 Training times **19**

Supplementary Note 1. Fragment correction scheme

To address non-covalently bound structures separated by distances larger than the molecular descriptor cutoff, we introduce a fragment correction scheme, illustrated in Figure 1. Before making a prediction for a given system, the method first checks for non-covalently bound fragments. Predictions are then obtained both for the full system and for each individual fragment. If the excitation energy of the total system is higher than the lowest excitation energy among the fragments, this indicates that OMNI-P2x has likely treated the system as multiply excited, with separate excitations localized on each chromophore. In such cases, the fragment-based results are used and combined to construct the final spectrum. Conversely, if the excitation energy of the total system is lower than the excitation energies of all subsystems, the state is identified as an excimer, and the total-system result is retained.

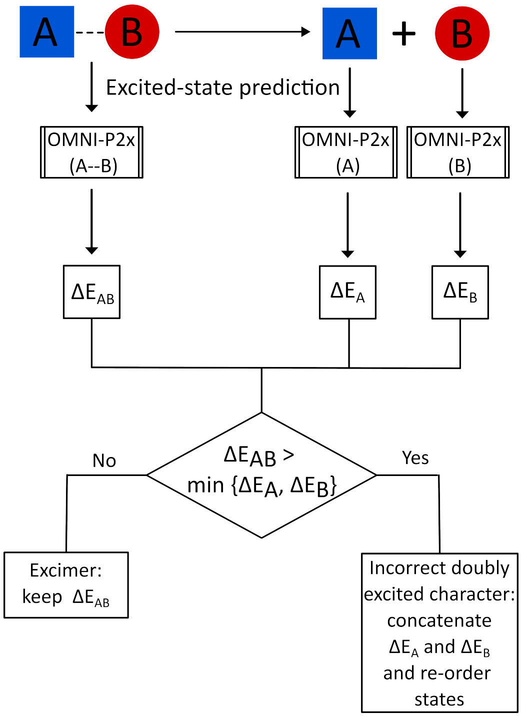

Supplementary Figure 1: Schematic depiction of the fragment correction scheme used in this work.

To validate this scheme, we computed the first excitation energy of a benzene dimer as a function of the intermolecular separation R, with results shown in Figure 2. We compare corrected and uncorrected OMNI-P2x predictions with reference TD-DFT/B3LYP. While the uncorrected model

initially gives results reasonably close to TD-DFT (despite the absence of dimeric systems in the training set), its predictions diverge for R between 3 and 4 Å: as parts of the system fall outside the cutoff radius, the excitation energy increases and approaches nearly twice the TD-DFT value. In contrast, the corrected model yields results in excellent agreement with the reference above the descriptor cutoff.

It should also be noted that TD-DFT itself is not the most reliable method for systems with large spatial separations, as its well-known inability to reproduce the hydrogen dissociation curve exemplifies a broader limitation of density functional theory. This is reflected here by an unphysical bump in the excitation energy between 8 and 9 Å.

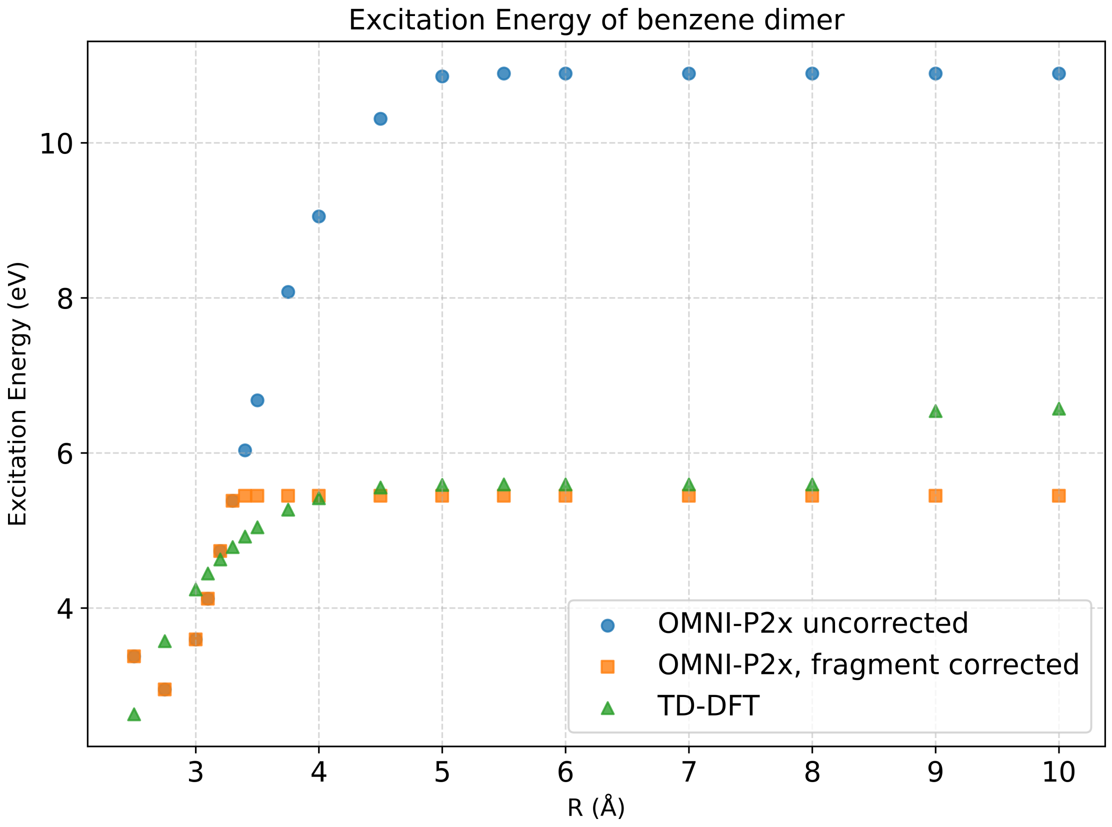

Supplementary Figure 2: First excitation energy of benzene dimer as a function of the separation R. Green triangles are reference, TD-DFT results, blue circles represent uncorrected OMNI-P2x predictions, while orange squares represent fragment-corrected OMNI-P2x.

At the same time, fine-tuning of the OMNI-P2x model on structures of benzene dimers allows it to correctly replicate the relationship between excitation energy and monomer separation.

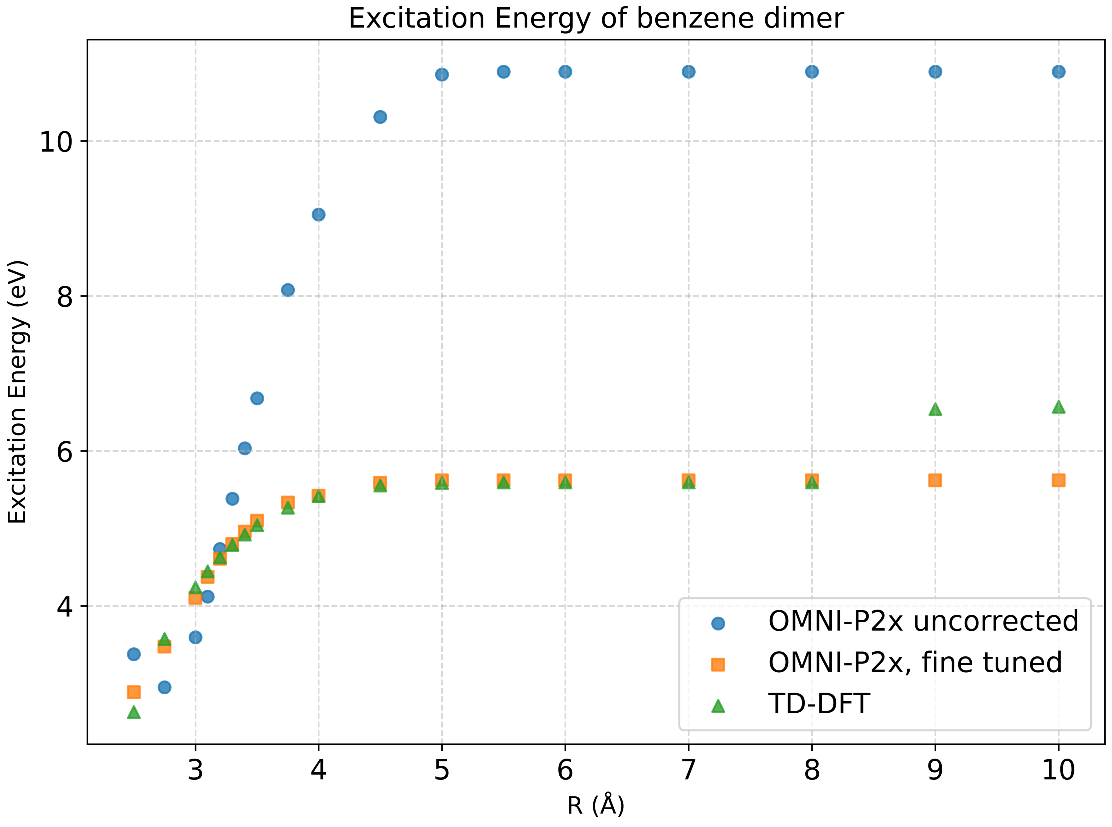

Supplementary Figure 3: First excitation energy of benzene dimer as a function of the separation R. Green triangles are reference, TD-DFT results, blue circles represent uncorrected OMNI-P2x predictions, while orange squares represent fine-tuned OMNI-P2x.

Supplementary Note 2. Performance of OMNI-P2x

2.1 Error statistics of excitation energies

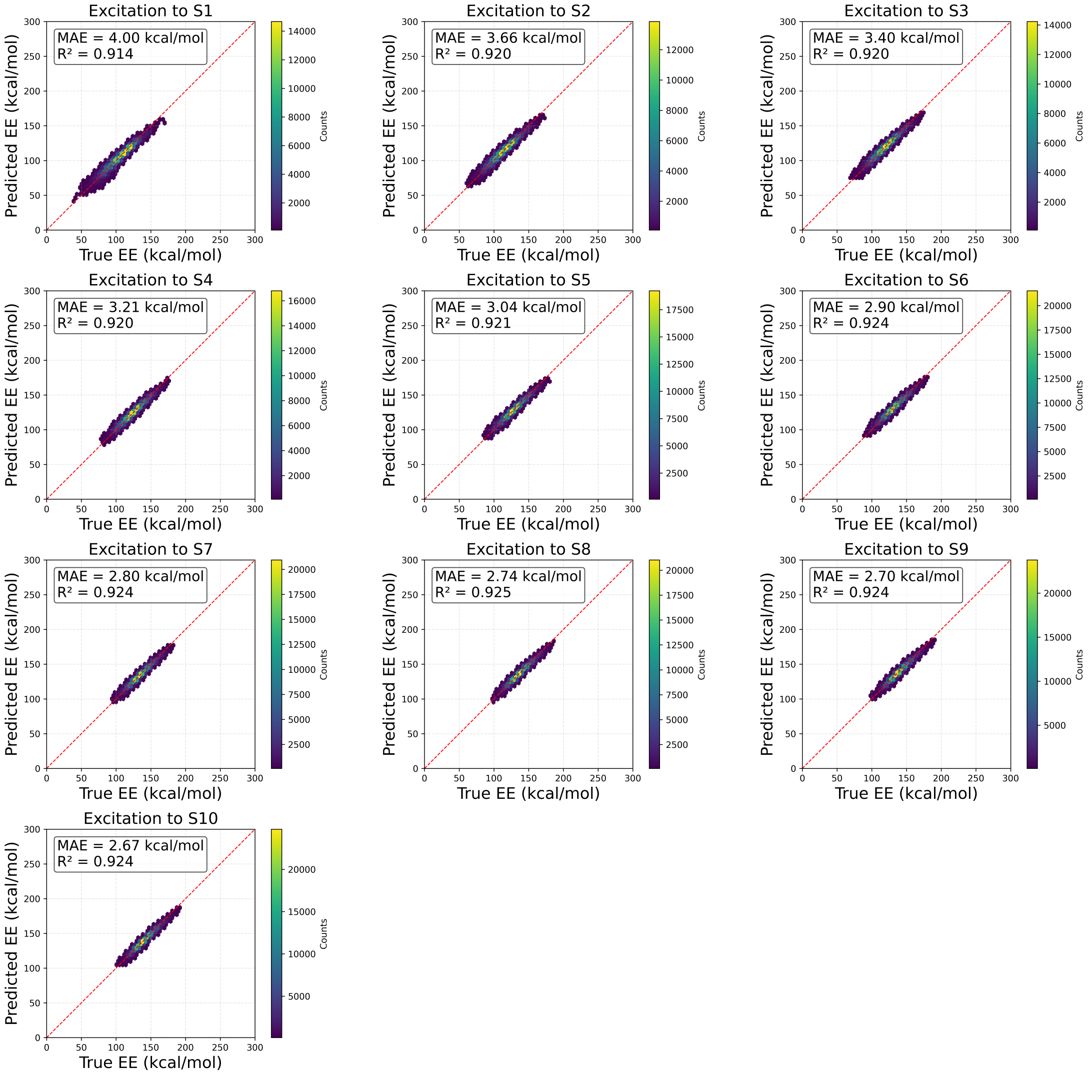

Supplementary Figure 4: Correlation plots of predicted vs true excitation energies for OMNI-P2x, for each electronic states, along with the R^2 metric and MAE.

2.2 Predicted spectra

For the following analysis, the spectra were ranked by a geometric mean of normalized MAE's for the excitation energy and oscillator strength, which for a given molecule k has the form:

$$S^{(k)} = \sqrt{\mathrm{MAE}_E^{(k)} \cdot \mathrm{MAE}_f^{(k)}} \tag{S1}$$

where:

$$\mathrm{MAE}_E^{(k)} = \frac{\frac{1}{N_{\mathrm{exc}}} \sum_{i=1}^{N_{\mathrm{exc}}} \left| E_i^{(k)} - \hat{E}_i^{(k)} \right|}{E_{\max}^{(k)} - E_{\min}^{(k)}}, \tag{S2}$$

$$\mathrm{MAE}_f^{(k)} = \frac{\frac{1}{N_{\mathrm{exc}}} \sum_{i=1}^{N_{\mathrm{exc}}} \left| f_i^{(k)} - \hat{f}_i^{(k)} \right|}{\sum_{i=1}^{N_{\mathrm{exc}}} f_i^{(k)}}. \tag{S3}$$

Which corresponds to normalizing the errors of the excitation energies by the spectral range $(E_{\max}^{(k)} - E_{\min}^{(k)})$ and the oscillator strengths by their sum $(\sum_{i=1}^{N_{\mathrm{exc}}} f_i^{(k)})$, for a given molecule.

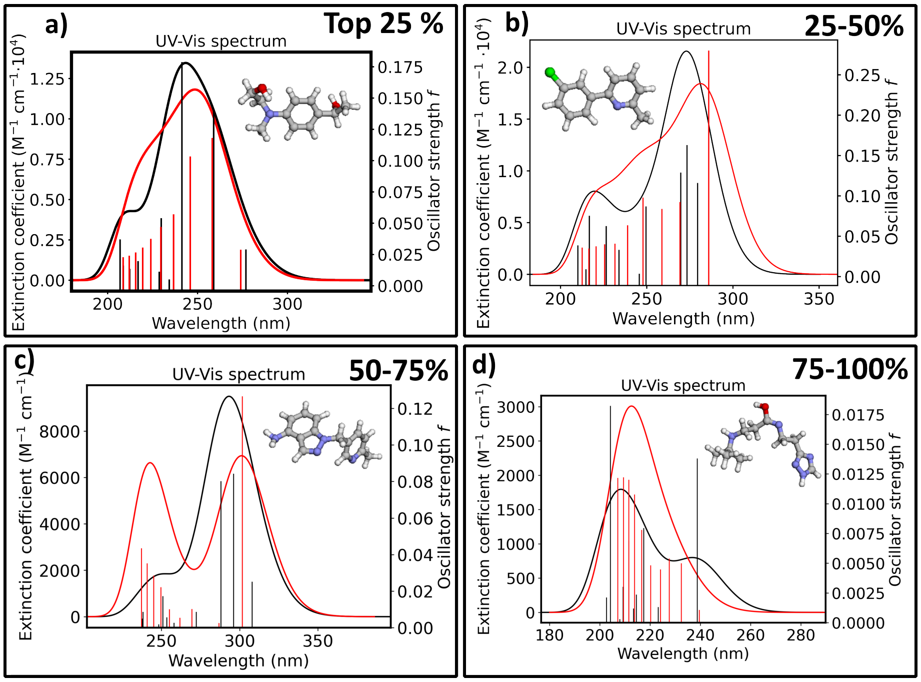

Supplementary Figure 5: Comparison between reference TD-DFT/B3LYP spectra (black lines) and OMNI-P2x predicted spectra (red lines), ranked by excitation energy and oscillator strength error score. Four examples are shown, randomly chosen from the top 25% of the predicted spectra (panel a), 25–50% (panel b), 50–75% (panel c) and bottom 25% (panel d).

2.3 Non-equilibrium structures

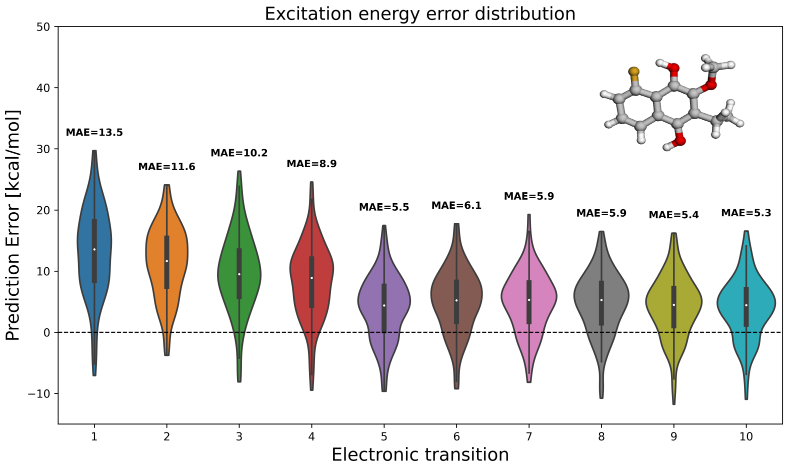

Supplementary Figure 6: Violin plot showing the error distribution of excitation energies predicted on thermally-distorted non-equilibrium structures of the top 25% molecule presented in figure 5 of the main text.

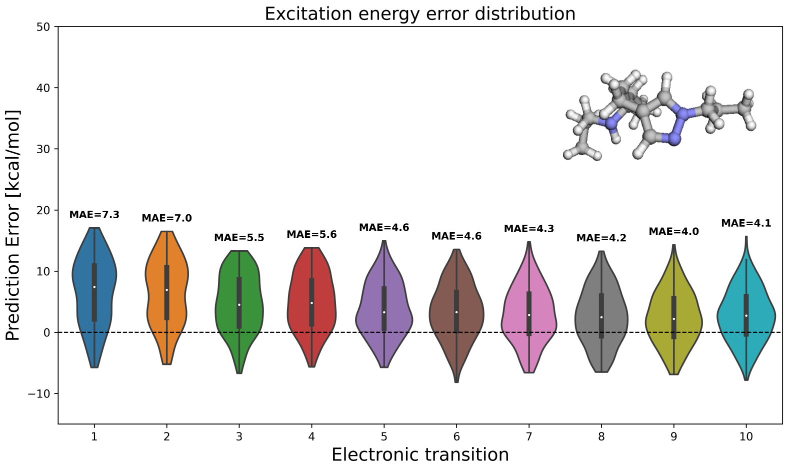

Supplementary Figure 7: Violin plot showing the error distribution of excitation energies predicted on thermally-distorted non-equilibrium structures of the second quartile molecule presented in figure 5 of the main text.

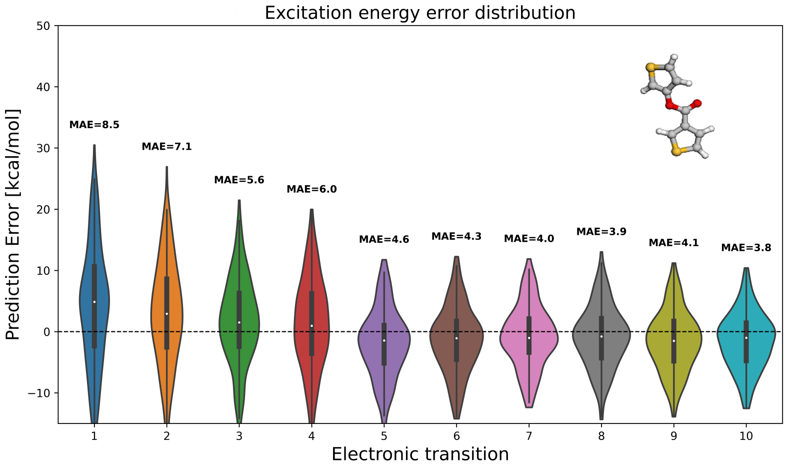

Supplementary Figure 8: Violin plot showing the error distribution of excitation energies predicted on thermally-distorted non-equilibrium structures of the third quartile molecule presented in figure 5 of the main text.

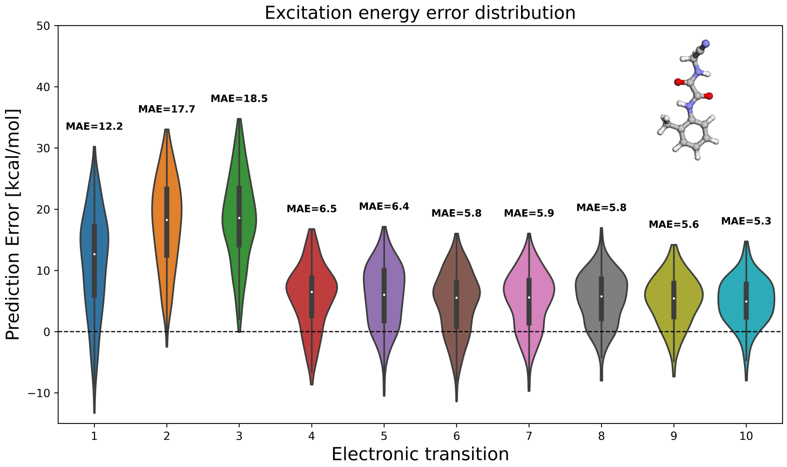

Supplementary Figure 9: Violin plot showing the error distribution of excitation energies predicted on thermally-distorted non-equilibrium structures of the bottom quartile molecule presented in figure 5 of the main text.

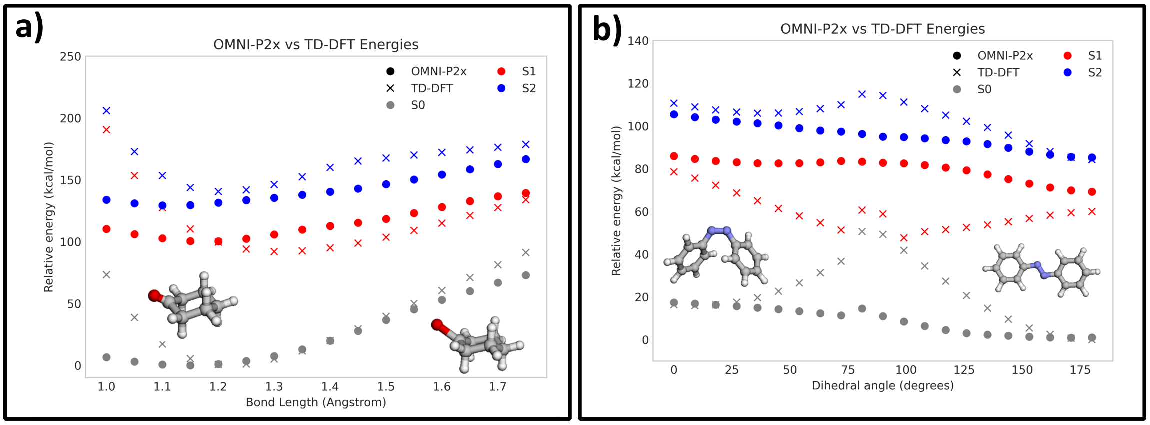

Supplementary Figure 10: Relaxed potential energy surface scans of (a) cyclohexanone along the C=O bond stretch and (b) azobenzene along the C–N=N–C torsion. Geometries were optimized at the DFT/B3LYP(6-31+G*) level, and excitation energies were computed with TD-DFT and OMNI-P2x (DFT-level predictions) for the lowest three states.

Supplementary Note 3. High-throughput screening of azobenzene derivatives

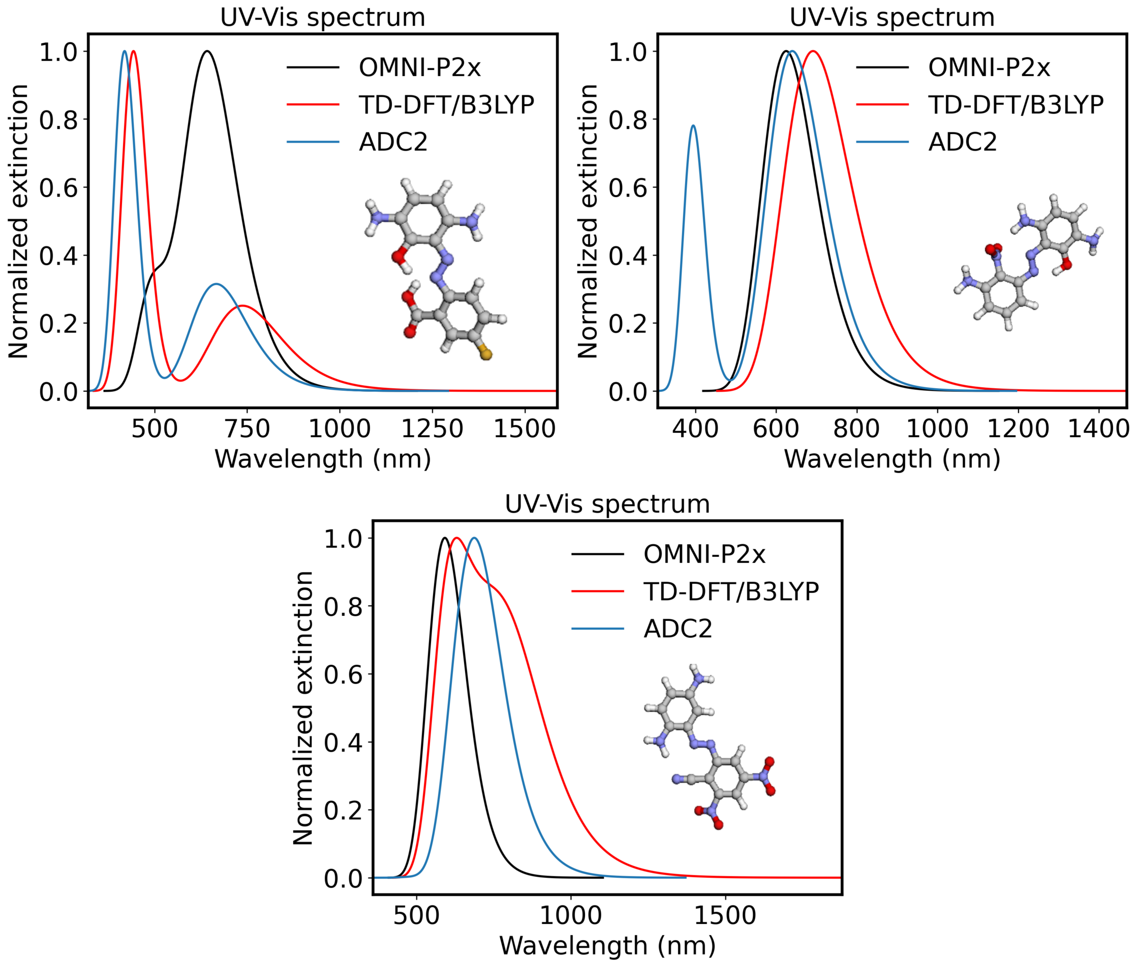

Supplementary Figure 11: Comparison of the spectra predicted using OMNI-P2x, TD-DFT and ADC(2), for the top 3 screened azobenzene derivatives.

Supplementary Note 4. Nonadiabatic molecular dynamics

4.1 Fulvene

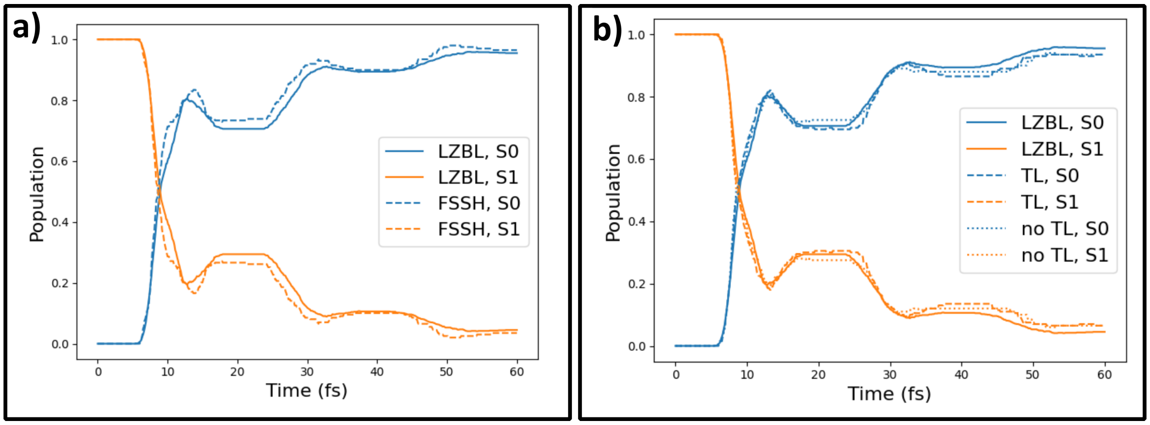

Supplementary Figure 12: Comparison of electronic state populations of fulvene in dynamics propagated with two different trajectory surface hopping schemes – FSSH and LZBL without kinetic energy reduction (panel a); comparison of reference LZBL dynamics without kinetic energy reduction with populations predicted by TL and no TL models (panel b).

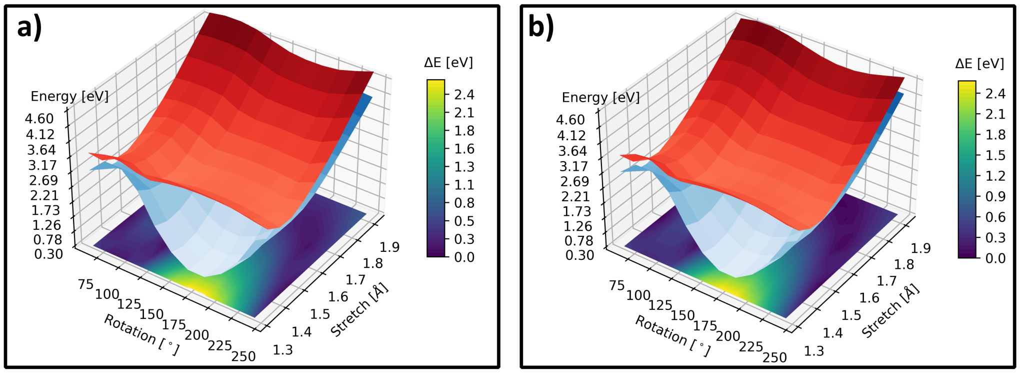

Supplementary Figure 13: Comparison of the S_0 and S_1 PES' as predicted by the transfer learning ML model (a); reference CASSCF (6,6) calculations (b). The PES is spanned by the two key reaction coordinates for fulvene, C=CH2 stretching and rotation.

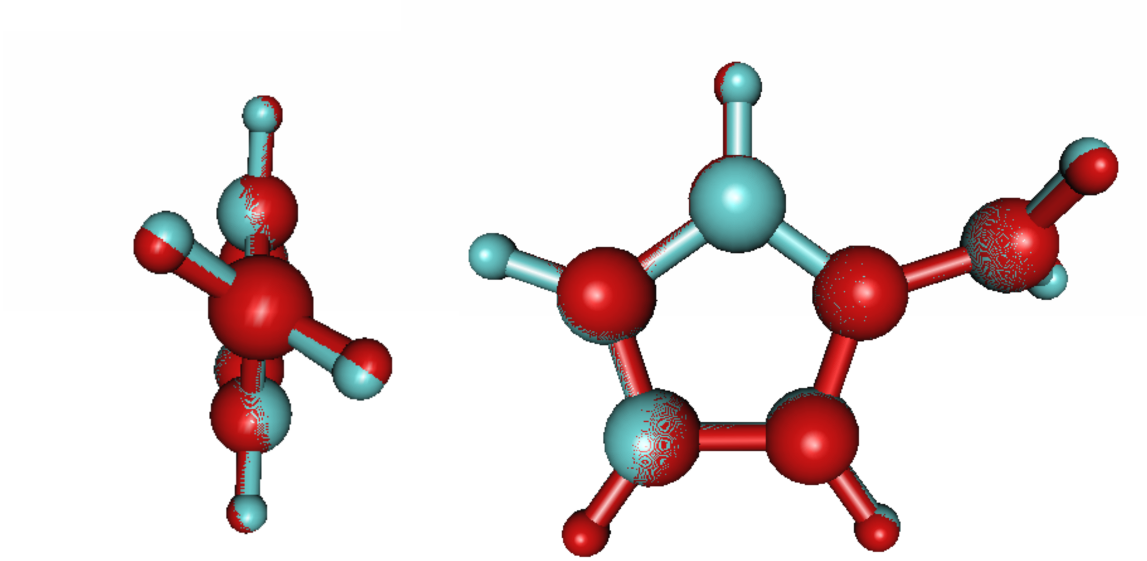

Supplementary Figure 14: Overlap between the ML-optimized and CASSCF-optimized MECI, for fulvene. The RMSD between the two structures is equal to 0.03 Å.

Supplementary Table 1: Key parameters describing the conical intersection optimized using the ML model, and CASSCF(6,6).

Parameter	OMNI-P2x-TL@CASSCF(6,6)	CASSCF (6,6)
Mean C=CH2 dihedral [degrees]	90.51	90.03
C=CH2 distance [A]	1.477	1.479

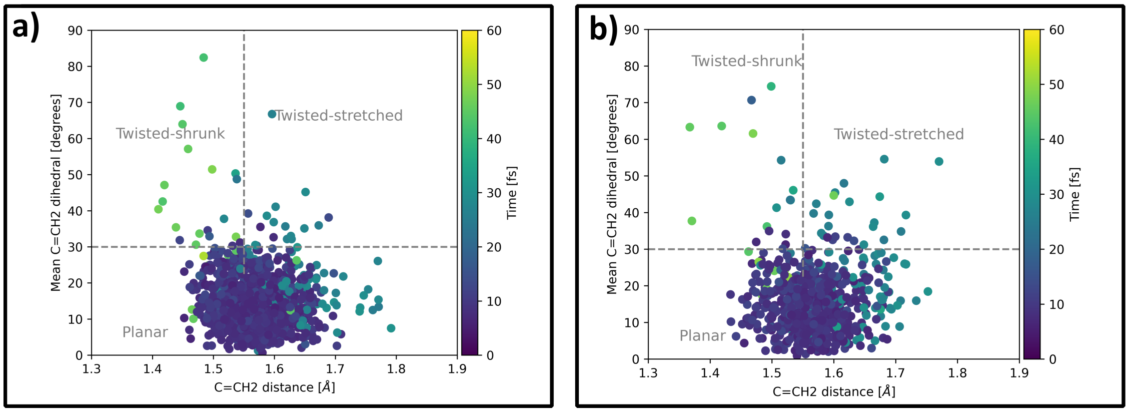

Supplementary Figure 15: Correlation plots of the C=CH2 distance and the mean dihedral angle at the $S_1 \rightarrow S_0$ hopping points in ML-TSH dynamics (a) and reference CASSCF dynamics (b).

Supplementary Table 2: Mean value and error bars (95% confidence interval) of observables describing the deactivation channels of fulvene for ML dynamics and reference CASSCF dynamics.

Observable	CASSCF dynamics	ML dynamics
Planar hopping (%)	94.0 ± 1.8	96.3 ± 1.1
Twisted-stretched hopping (%)	3.7 ± 1.5	1.7 ± 0.8
Twisted-shrunk hopping (%)	2.3 ± 1.2	2.0 ± 0.8

4.2 Azobenzene

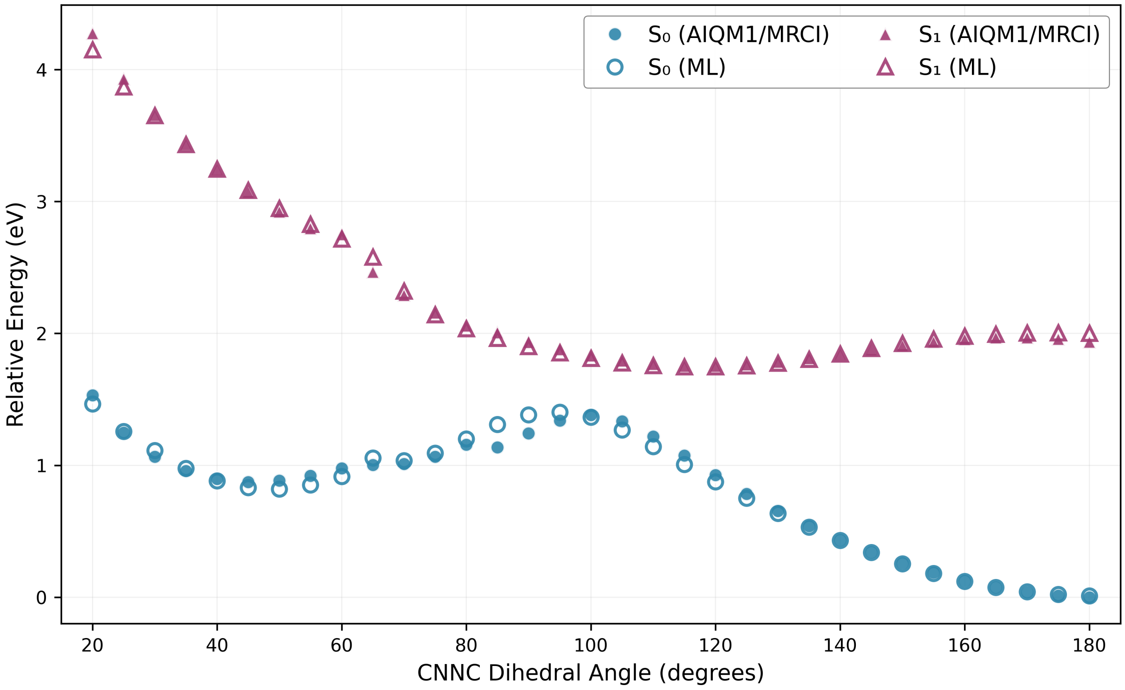

Supplementary Figure 16: Comparison of the azobenzene PES with respect to central bond torsion, obtained with reference AIQM1/MRCI calculations (full symbols), and the ML model (empty symbols), for S_0 and S_1.

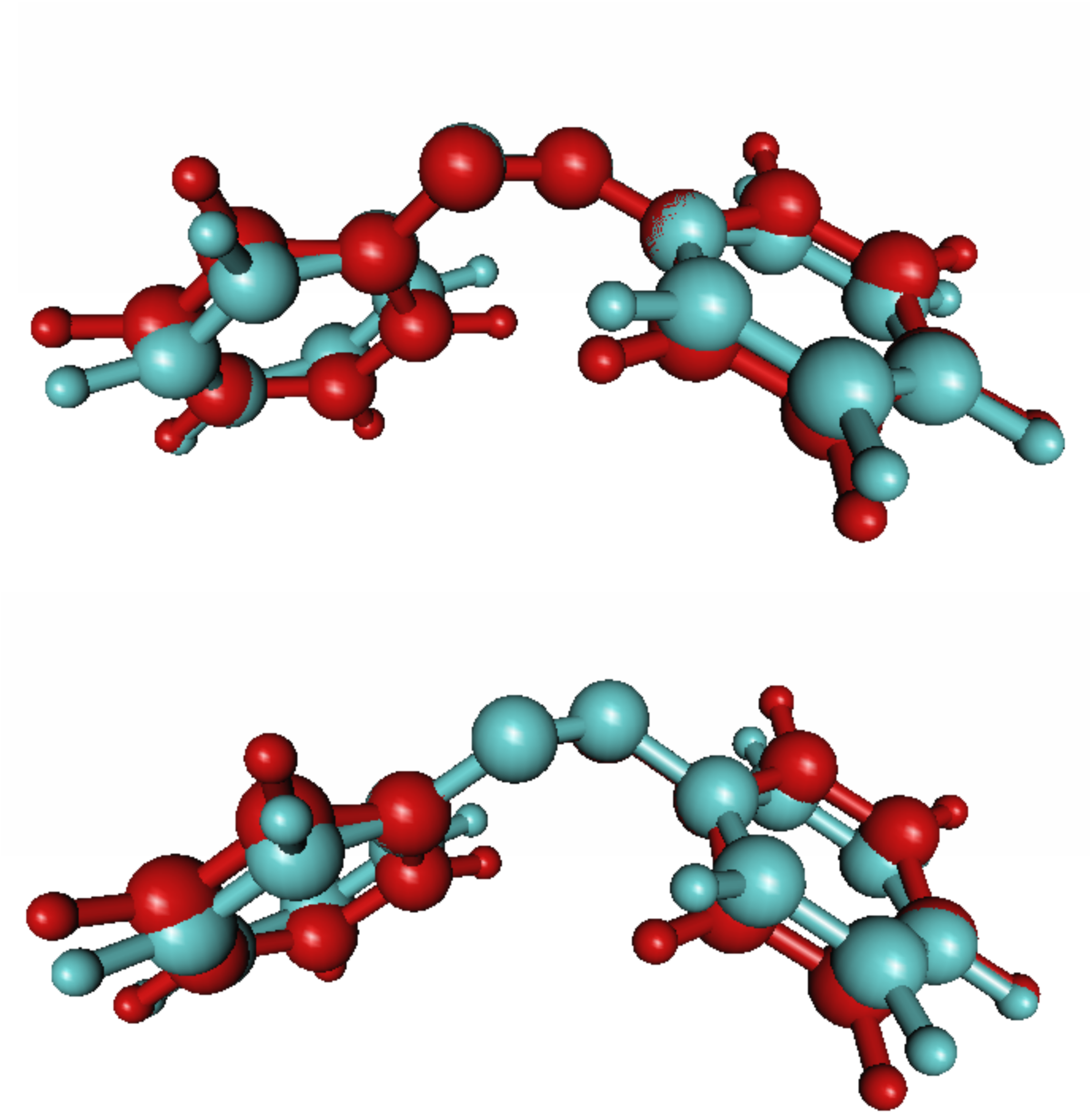

,

Supplementary Figure 17: Overlap between the ML-optimized and AIQM1-MRCI optimized MECI for azobenzene.

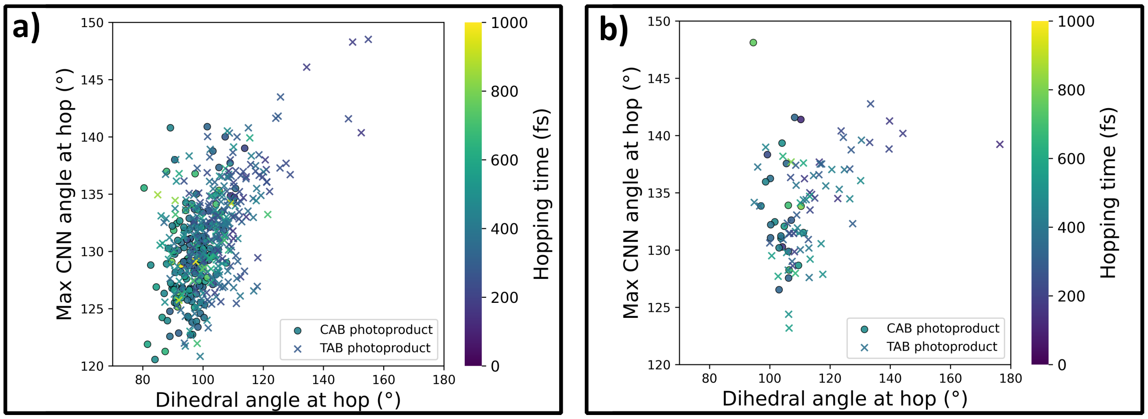

Supplementary Figure 18: Correlation plots of the maximum C-N=N angle and the C-N=N-C dihedral angle of azobenzene at the $S_1 \rightarrow S_0$ hopping points in ML-TSH dynamics (a) and reference AIQM1/MRCI dynamics (b).

Supplementary Note 5. Performance of B3LYP

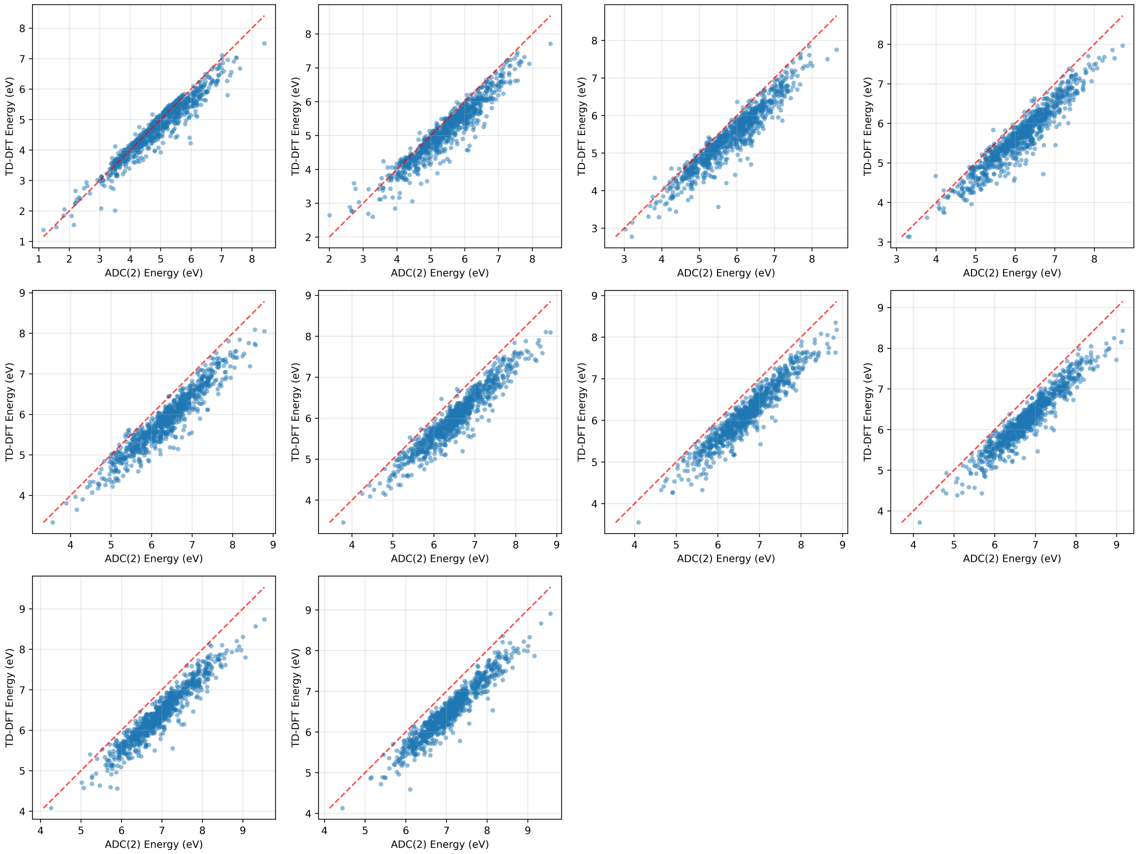

Supplementary Figure 19: Correlation plots between ADC2 and TD-DFT/B3LYP excitation energies for 1000 molecules randomly selected from the PubChemQC dataset.

Supplementary Table 3: Statistical parameters of the correlation between ADC(2) and TD-DFT/B3LYP excitation energies: the Pearson's correlation coefficient R, mean absolute error (MAE) and mean signed error (MSE).

Target state	R	MAE (eV)	MSE (eV)
S1	0.9598	0.24	-0.18
S2	0.9493	0.34	-0.32
S3	0.9488	0.42	-0.41
S4	0.9479	0.46	-0.46
S5	0.9488	0.50	-0.50
S6	0.9513	0.52	-0.52
S7	0.9514	0.54	-0.54
S8	0.9513	0.55	-0.55
S9	0.9520	0.56	-0.56
S10	0.9560	0.57	-0.57

Supplementary Note 6. Training times

The 4 main models of OMNI-P2x (ensemble of three energy-predicting NNs and oscillator strength NN) have been trained for 100 epochs on 32 NVIDIA GH200 GPUs with 96GB memory each, which totaled to about 48 hours of training time per model. Fine tuning of OMNI-P2x for NEA spectra was performed in a *Jupyter Lab* environment, with 4 Intel Xeon Gold 6226R CPU cores, taking under a minute for the pyrazine case, and about 40 minutes for 9-DCMA. The AL loop for NAMD simulations of fulvene was ran on a computational node with a single NVIDIA 4090 RTX GPU and 16 Intel Xeon Gold 6226R CPUs, taking 24h to converge. Fine-tuning of OMNI-P2x for NAMD simulations was performed with the same computational resources, taking about 20 minutes per trained model.
